# Positive Selection of *Iris,* a Retroviral *Envelope*–Derived Host Gene in *Drosophila melanogaster*

**DOI:** 10.1371/journal.pgen.0010044

**Published:** 2005-10-21

**Authors:** Harmit S Malik, Steven Henikoff

**Affiliations:** 1 Basic Sciences Division, Fred Hutchinson Cancer Research Center, Seattle, Washington, United States of America; 2 Howard Hughes Medical Institute, Fred Hutchinson Cancer Research Center, Seattle, Washington, United States of America; Stanford University School of Medicine, United States of America

## Abstract

Eukaryotic genomes can usurp enzymatic functions encoded by mobile elements for their own use. A particularly interesting kind of acquisition involves the domestication of retroviral *envelope* genes, which confer infectious membrane-fusion ability to retroviruses. So far, these examples have been limited to vertebrate genomes, including primates where the domesticated *envelope* is under purifying selection to assist placental function. Here, we show that in *Drosophila* genomes, a previously unannotated gene *(CG4715,* renamed *Iris)* was domesticated from a novel, active *Kanga* lineage of insect retroviruses at least 25 million years ago, and has since been maintained as a host gene that is expressed in all adult tissues. *Iris* and the *envelope* genes from *Kanga* retroviruses are homologous to those found in insect baculoviruses and *gypsy* and *roo* insect retroviruses. Two separate *envelope* domestications from the *Kanga* and *roo* retroviruses have taken place, in fruit fly and mosquito genomes, respectively. Whereas retroviral envelopes are proteolytically cleaved into the ligand-interaction and membrane-fusion domains, *Iris* appears to lack this cleavage site. In the *takahashii/suzukii* species groups of *Drosophila,* we find that *Iris* has tandemly duplicated to give rise to two genes *(Iris-A* and *Iris-B). Iris-B* has significantly diverged from the *Iris-A* lineage, primarily because of the “invention” of an intron de novo in what was previously exonic sequence. Unlike domesticated retroviral *envelope* genes in mammals, we find that *Iris* has been subject to strong positive selection between *Drosophila* species. The rapid, adaptive evolution of *Iris* is sufficient to unambiguously distinguish the phylogenies of three closely related sibling species of *Drosophila (D. simulans, D. sechellia,* and *D. mauritiana),* a discriminative power previously described only for a putative “speciation gene.” *Iris* represents the first instance of a retroviral *envelope*–derived host gene outside vertebrates. It is also the first example of a retroviral *envelope* gene that has been found to be subject to positive selection following its domestication. The unusual selective pressures acting on *Iris* suggest that it is an active participant in an ongoing genetic conflict. We propose a model in which *Iris* has “switched sides,” having been recruited by host genomes to combat baculoviruses and retroviruses, which employ homologous *envelope* genes to mediate infection.

## Synopsis

Mobile genetic elements have made homes within eukaryotic (host) genomes for hundreds of millions of years. These include retroviruses that integrate into host genomes as an essential step in their life cycle. While most such integration events are likely to be either deleterious or of little consequence to the host, on rare occasions host genomes can preserve and exploit capabilities of mobile elements for their own function. Especially intriguing are instances where host genomes have chosen to retain the *envelope* genes of retroviruses; the same *envelope* genes are responsible for conferring infectious ability to retroviruses. Primates and rodent genomes each have domesticated retroviral *envelope* genes (called *“syncytin”* genes) for important roles in placental function.

Now, Harmit Malik and colleagues show that a similar, ancient domestication event has taken place within the fruit fly *Drosophila melanogaster*. They identify a gene, *Iris*, which was acquired from an *envelope* gene of insect retroviruses, and has been maintained as a host gene for more than 25 million years. Unexpectedly, the authors find that *Iris* continues to evolve rapidly whereas previous studies have shown that mammalian *syncytin* genes do not. They suggest a model in which the *Iris* gene has “switched sides,” from its original role in causing infections to its current role in preventing them.

## Introduction

Despite the fact that mobile elements are generally detrimental to host fitness, there are several instances where eukaryotic genomes have harnessed the enzymatic machinery of transposable elements to perform a myriad of important functions. For instance, the reverse transcriptase activity of the telomerase enzyme, which protects the ends of linear chromosomes [1], is believed to be the ancient descendant of prokaryotic mobile genetic elements [2]. In several species of *Drosophila,* active Het-A and TART retroposons still carry out this important function [3,4]. The core enzymatic machinery used to carry out V(D)J recombination in the generation of antigen recognition diversity is encoded by the RAG1/RAG2 proteins, believed to be descended from a previously autonomous transposon [5,6]. Many human genes are derived from the integrase machinery of transposable elements [7–9], and although their function is still unknown, many of them appear to have conserved their enzymatic ability [10]. Host genomes can also employ mobile elements' genes for genome defense. In murine genomes, a domesticated retroviral *gag* gene, *Fv1,* can defend mouse cells against infections by exogenous retroviruses [11]. These represent examples of how host genomes can acquire and eventually exploit the enzymatic capabilities of mobile elements for host functions.

“Domestication” of retroviral *envelope (env)* genes is especially intriguing in this context. While the *env* gene usually confers infectious ability to retroviruses, the human endogenous retrovirus-W *env* gene now appears to play a critical role in placental morphogenesis in higher primate genomes [12]. This gene, called *syncytin,* is still present in the context of a human endogenous retrovirus-W provirus that entered the primate lineage about 35 million years ago [13], indicating that it is still at the early stages of “evolutionary domestication” in its transition from a retroviral *env* to a host gene [14,15]. Indeed, selection pressures on the rest of the retroviral sequence show early signs of decay, but the *syncytin* gene itself is under strong selective constraints and is conserved among all hominoids and Old World monkeys [14]. Thus, while the endogenous retrovirus itself has lost the service of its *env* gene, host genomes now exploit this gene's membrane-fusion ability to carry out the important process of trophoblast differentiation [12,16]. Recently, three other retrovirus *env*-derived host genes have been described. *Syncytin-2* is a 35-million-year-old host gene also found in primate genomes, which is derived from human endogenous retrovirus-FRD and appears to be predominantly expressed in placenta [17]. Two separate retrovirus-derived fusogenic *env* genes, *syncytin-A* and *syncytin-B,* have been shown to be expressed in murine placental tissues [18]. These genes represent a remarkable case of convergent evolution where rodent and primate genomes have each acquired retroviral *env* genes for important roles in placental differentiation.

Most retroviruses appear to be derived from ancestral non-viral retrotransposons that lacked infectious ability [19,20]. Phylogenetic analysis suggests that the acquisition of *env* genes drove the evolutionarily important transition from a non-viral retrotransposable element to an infectious retrovirus on at least nine occasions [20,21]. Two of these instances led to the *gypsy* and *roo* retroviruses in *Drosophila,* which have both separately acquired homologous *env* genes from baculoviruses, double-stranded DNA viruses with large genomes [20,22]. Many baculoviruses employ this *env* gene for mediating infection [23]. In both retroviruses and baculoviruses, infectious ability requires a proteolytic cleavage to separate the envelope protein into the SU (receptor-binding component) and TM (brings membranes into close apposition and causes fusion) proteins. Just downstream of furin cleavage site is a hydrophobic fusion peptide that is also required for membrane fusion [24,25].

The release of the *D. melanogaster* genome sequence [26] provided a unique resource to help address the chronology of *env* acquisition by retroviruses. For instance, it gave a sequence snapshot of all proviral insertions in the *D. melanogaster* genome [27,28]. Compared to mammalian genomes, *Drosophila* genomes have a higher rate of DNA loss [29], thus proviral sequences are more likely to reflect recent insertion events or insertions that have been selectively retained. In our survey, we unexpectedly found that the *D. melanogaster* genome contains a host gene, *CG4715* (renamed *Iris* in this paper), which is homologous to the *env* genes from baculoviruses and insect retroviruses (also identified in [22] [30]). We have now investigated the evolution of *Iris* in insect genomes, and found it to be conserved in most *Drosophila* species of the *Sophophora* subgenus. We can trace the acquisition of this *env* gene to a sister lineage of the *roo* insect retroviruses (named *Kanga* in this paper). Investigation of the selective constraints on *Iris* reveals that it has been subject to positive selection throughout its evolution in *Drosophila.* This unusual finding of positive selection on a domesticated retroviral *env* gene suggests that it is an active participant in an extant genetic conflict in its host genomes, possibly to combat against insect viruses that bear homologous *env* genes.

## Results

### *CG4715* is a Viral *Envelope*–Related Host Gene in *Drosophila*

**Figure 1:**
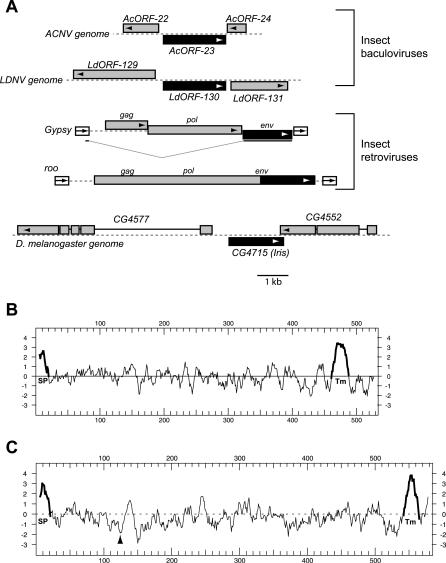
*CG4715* Homologs (A) Baculoviral and insect retroviral *env* genes shown in their respective genomic context. Baculoviruses, represented by *Autographa californica nucleopolyhedrovirus* (ACNV) and *Lymantria dispar nucleopolyhedrosis virus* (LDNV) are double-stranded DNA viruses whose genome size is close to 150 kilobases [72], while retroviruses, represented by *roo* and *Gypsy,* are close to 7 kilobases in length [73]. *CG4715* is an open reading frame found in the same genomic context in many species of *Drosophila*. *CG4715/Iris* and its *env* homologs are shown in black (open reading frame direction shown by arrows) while neighboring genes are shown in gray. Note that the *gypsy env* is expressed through a spliced message. Kyte-Doolittle hydropathy plots of encoded protein products from *CG4715* (B) and the *roo env* gene (C) are shown. The putative signal peptide (SP) and C-terminal, transmembrane hydrophobic peptide (Tm) are highlighted in bold, while the furin cleavage site in the *roo* envelope protein is indicated by an arrowhead.

In order to investigate whether or not the *D. melanogaster* genome had domesticated any retroviral genes, we initiated searches of the databases by PSI-BLAST using the various encoded genes from the *gypsy* and *roo* insect retroviruses. We found a strong match to their *env* genes in a previously unannotated gene, *CG4715,* in the *D. melanogaster* genome [22]. The genomic regions surrounding *CG4715* bear no discernible similarity to baculoviral or retroviral sequences, ruling out the possibility that *CG4715* represents the evolutionary remnant of a recent retroviral-introduced provirus or a baculoviral insertion. Figure 1A schematizes the genomic contexts of the *env* homologs found in baculoviral, retroviral, and the *D. melanogaster* genomes. *CG4715* bears many of the hallmarks of the *gypsy* and *roo env* genes, including the same architecture consisting of a signal peptide and a carboxyl-terminal hydrophobic peptide that is likely to be membrane-spanning (Figure 1B and 1C).

**Figure 2:**
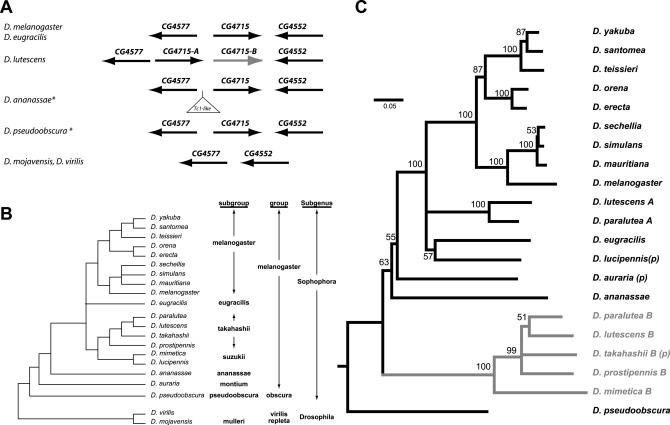
Phylogenetic Analysis of *CG4715* Homologs (A) *CG4715* has been preserved in its syntenic location in *Drosophila* species. In species from the *takahashii/suzukii* species groups like *D. lutescens,* an additional paralog, *CG4715-B* (gray shading) is found in tandem orientation. *D. ananassae* has an additional transposon insertion in this syntenic location between *CG4715* and *CG4552,* while the genomes of *D. mojavensis* and *D. virilis* lack *CG4715* orthologs between *CG4577* and *CG4552*. For *D. ananassae* and *D. pseudoobscura,* sequence was obtained from genome sequencing data (indicated with an asterisk) and confirmed by sequencing. (B) An “expected” phylogeny of *Drosophila* species is shown, summarizing results from many genes [30,31]. (C) A neighbor-joining phylogeny of *CG4715* orthologs based on C-terminal amino acid sequence is presented. (For some species, only the C-terminal sequence was obtained (indicated by a “p” for partial)). This phylogeny is largely in agreement with the accepted species phylogeny in (B), indicating that the gene has been inherited by strict vertical inheritance. Although there is a slight discordance in phylogenetic placement of the *D. ananassae, D. eugracilis,* and *D. auraria,* these branches have only a low bootstrap support. A second lineage of *CG4715* paralogs, *CG4715-B* is evident (gray shading) in the *takahashii/suzukii* species groups.

We obtained *CG4715* sequence from ongoing genome sequencing projects in several species of *Drosophila* using synteny (gene order) and TBLASTN searches. We screened for the presence of *CG4715* in closely related species of the *Sophophora* subgenus of *Drosophila* using PCR and primers designed to flanking sequences (see Materials and Methods), and were able to confirm the presence of *CG4715* in several additional species of *Drosophila* (Figure 2A and 2C). During our sequencing efforts, we uncovered two *CG4715*-related genes in tandem orientation in all species of the *takahashii/suzukii* subgroups. Figure 2C represents the phylogenetic analysis of *CG4715* genes in the *Sophophora* subgenus of *Drosophila* (based on a partial alignment of their coding sequences), whose phylogenetic relationship is in good agreement with the widely accepted phylogeny of this genus [31,32] (schematized in Figure 2B). This indicates that this gene has been inherited strictly by vertical inheritance rather than by horizontal transfer, a conclusion that is supported by the fact that *CG4715* is found in the same syntenic location in different species (Figure 2A). Of the two *CG4715* genes found in the *takahashii/suzukii* groups, the 5′ gene (referred to as *CG4715-A*) represents the true ortholog, while the second *(CG4715-B)* represents a gene duplication whose phylogenetic position (Figure 2C) is incongruent with the expected species phylogeny (schematized in Figure 2B). This phylogenetic placement could result from altered selective constraints (and different evolutionary rates) that could lead to a phylogenetic artifact known as “long-branch attraction” [33]. While we cannot rule out an ancient origin of the B lineage, this would lead to the unparsimonious implication that this gene was subsequently lost in all species except those from the *takahashii/suzukii* species groups.

In *D. mojavensis* and *D. virilis,* whose genome sequences are still incomplete, *CG4715* is absent from its syntenic location, and we have not found true orthologs in other genomic locations. While it remains formally possible that the location of *CG4715* is altered in these two species, it is more likely that *CG4715* does not exist as a host gene in these species (BLAST searches did not reveal any orthologs). The latter possibility could be due to a subsequent loss event in *D. mojavensis and D. virilis* (both belong to the *Drosophila* subgenus, Figure 2B) or because *CG4715* originated only after the separation of the *Sophophora* and *Drosophila* subgenera. Completion of ongoing sequencing projects in the *D. willistoni, D. mojavensis, D. virilis,* and *D. grimshawi* species will help distinguish among these possibilities.

### *CG4715* is the Domesticated *Envelope* Gene of the *Kanga* Insect Retroviruses

**Figure 3:**
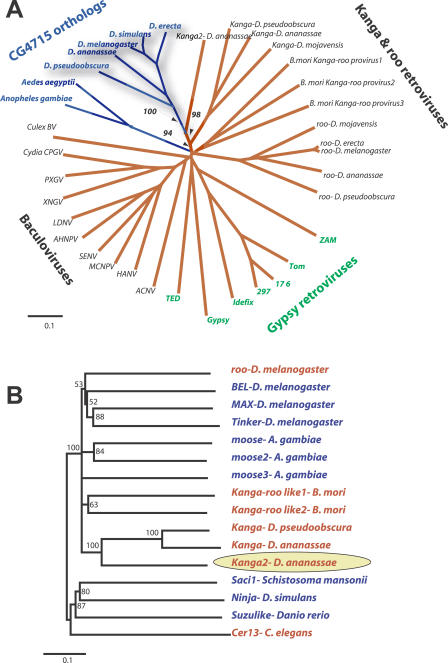
*CG4715*/*Iris* Relationships to Viral *Envelope* Genes (A) The central domains of *CG4715* and related viral *env* genes were aligned, and a neighbor-joining phylogenetic tree constructed. The tree separates the *CG4715-env* superfamily into four groups: the baculoviruses, the BEL clade retroviruses *roo* and *Kanga,* the *gypsy*-like retroviruses, and host genome borne *CG4715* orthologs in *Drosophila* and mosquito genomes. While the tree overall does not provide high resolution to discern the order of divergence of each of the clades, there is very strong phylogenetic resolution (bootstrap support of key nodes shown) to unambiguously group *CG4715* orthologs with the *Kanga* retrovirus lineage, indicating that this lineage of retroviruses is the likely source of the *CG4715* lineage. (B) Neighbor-joining phylogeny of selected representatives from the BEL clade of retrotransposons indicates that the *Kanga* retroviruses from *Drosophila* genomes form a monophyletic clade (the presumed ancestor of *CG4715* is indicated by a yellow oval). Most retrotransposons in the BEL lineage do not possess an *env* gene (blue lettering) while many elements that do (red) have acquired non-homologous *env* genes acquired from a different viral source [19,20].

Where did *CG4715* come from? The closest homologs to *CG4715* in the available sequences of all *Drosophila* genomes are the *env* genes of a novel lineage of retroviruses, which appear in several species of the *Sophophora* subgenus (ongoing sequencing projects, see Materials and Methods). This lineage of retroviruses is most closely related to the *roo* lineage of BEL-like retroviruses, and we refer to it as *Kanga* [34–36]. In a detailed phylogenetic analysis of all *CG4715-env* related genes (Figure 3A), the *CG4715* orthologs unambiguously branch together with the *env* genes of *Kanga*. We also investigated genome sequences from other insects for *CG4715* homologs. Remarkably, the *Anopheles gambiae* genome also contains a homolog of *CG4715* with the same architecture. Like the *Drosophila CG4715* gene, the *A. gambiae* gene is not flanked by regions homologous to either retroviral or baculoviral sequences. Using the *A. gambiae* gene as a query, we were able to successfully retrieve its *Aedes aegyptii* ortholog as well. We can detect *Kanga-roo*-like retroviruses in the lepidopteran *Bombyx mori* (silkmoth) genome, but not in the *Apis mellifera* (honeybee) genome. Intriguingly, while the *A. gambiae* genome has multiple retrotransposons related to the *Kanga-roo* retroviruses, none of these is predicted to encode an *env* gene. The primary reason that the *Kanga* retroviruses have not been described so far appears to be their absence in the earliest sequenced insect genomes, including *D. melanogaster* and *A. gambiae.*

On a phylogenetic tree of all *CG4715-env* related homologs (Figure 3A), the two mosquito genes represent a distinct lineage from that of the *Drosophila CG4715* orthologs and *Kanga* retroviruses. Parsimony criteria based on the phylogeny in Figure 3A strongly argues that the retroviral borne *env* gene represents the ancestral form. We can say with high confidence that the *Drosophila CG4715* genes have derived from within the *Kanga* retroviral lineage (bootstrap support on relevant nodes is highlighted in Figure 3A). Thus, we conclude that there have been two separate domestications of insect retroviral *env* genes in fruit fly and mosquito genomes, respectively. The domestication event in the *Sophophora* genus of *Drosophila* led to *CG4715,* which now has been preserved as a host gene. It is present in all species tested, and appears to have been inherited strictly by vertical inheritance for at least 25 million years (the estimated time of separation of *D. melanogaster* and *D. pseudoobscura* [31])*.*

To better gauge the evolutionary origins of the newly identified *Kanga* retroviruses, we compared the majority of the *pol* sequence (PR-RT-RNH domains) of *Kanga* to other known insect retrotransposon lineages. These showed that *Kanga* retroviruses belong unambiguously to the BEL clade, which also includes the *roo* but not the *gypsy* retroviruses (Figure 3B). Previous analyses have shown that only a few lineages of the BEL elements also possess *env* genes (red in Figure 3B) and that the *Caenorhabditis elegans* and *D. melanogaster* retroviruses of this lineage have non-homologous *env* genes[19,20], indicating that the non-infectious retrotransposons (blue lineages) are likely to be the ancestral form.

### Etymology

Based on our phylogenetic analyses (Figure 3A), it is clear that *CG4715* orthologs represent a sister lineage to the *env* genes from *Kanga* retroviruses. In Greek mythology, the Titan Thaumas and Electra had two sets of offspring. The first were the winged monsters, the Harpies (which we liken to the insect retroviral and baculoviral *env* genes). The second was Iris, the goddess of the rainbow and the messenger of the god Zeus and his wife, Hera. Since *CG4715* is clearly maintained as a host gene, we use the analogy to the benevolent sibling of the mythological Harpies to propose the name *Iris* to represent the *CG4715* orthologs, since they are related to viral *env* genes but are presumably beneficial to the host genome, based on their conservation.

### *Iris* Expression

**Figure 4:**
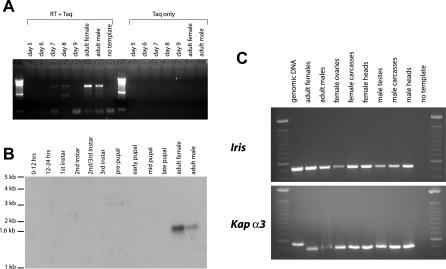
*Iris* Expression in *D. melanogaster* *Iris* expression through various stages of development was assayed using (A) RT-PCR and (B) Northern blots. Both show that *Iris* is predominantly expressed in adult females and males. (C) RT-PCR analysis on individually dissected tissues from adult flies shows that *Iris* is expressed in somatic tissues but expression is slightly reduced in ovaries and testes. RT-PCR to *Karyopherin alpha-3 (αKap3*, a ubiquitous nuclear import factor- *CG9423)* is shown as a control for amounts of template RNA in the RT-PCR reaction, and to show that there is no detectable contamination from genomic DNA.

Its strong conservation suggested that *Iris* might perform some important function in insects. To investigate this, we examined *Iris* expression in *D. melanogaster*. Using RT-PCR and Northern blots on pools of polyA RNA representing all life-stages of *D. melanogaster,* we determined that *Iris* is expressed primarily in adults in both males and females, with weak expression at the third instar larval stages (Figure 4A and 4B). By RT-PCR analysis on individually dissected tissues, we found *Iris* is transcribed in most adult tissues, with expression only slightly lower in ovaries and testes (Figure 4C). Our RT-PCR results are consistent with what was observed in a recent large-scale survey of *Drosophila* gene expression patterns in ovaries, testes, and the soma [37]. The expression pattern appears to suggest that *Iris* may have been domesticated for some role in adult flies, either within germline or somatic tissues, or both.

### Conserved Features among *Iris* Orthologs and Paralogs

**Figure 5:**
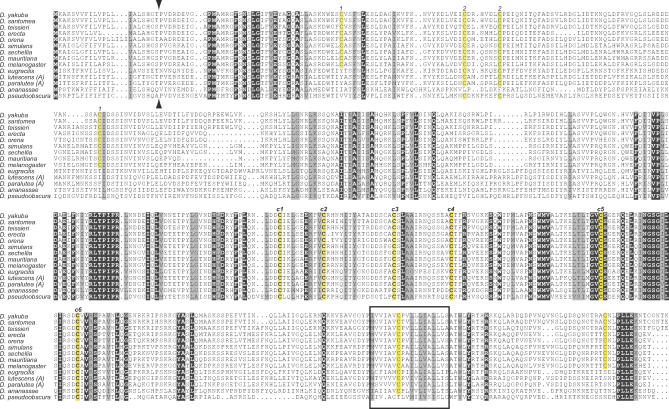
Complete Alignment of Iris Proteins An alignment of full-length Iris proteins from various *Drosophila* species is shown. All invariant residues are shown against a black background (except cysteines that are highlighted in yellow), while similar residues are highlighted in gray background. We did not include the *Iris-B* lineage here for ease of presentation (these are presented in Figure 6). Several features are conserved, including the signal peptide (predicted cleavage site indicated by arrowheads), C-terminal transmembrane domain (shown as a box), and several invariant cysteine residues (c1 through c6, highlighted in yellow) that are a characteristic feature of *Iris* and related envelope proteins. Other cysteine residue pairs (1–1 and 2–2, also highlighted in yellow) show co-conservation, i.e., loss of one results in loss of the other.

An amino acid alignment of all full-length *Iris* orthologs is shown in Figure 5. Several features are conserved, including a signal peptide (putative cleavage site shown by arrowheads) and a C-terminal hydrophobic peptide that presumably represents a membrane-spanning segment by analogy to the retroviral envelope proteins. In addition, several cysteine residues (highlighted in yellow) are variably conserved. Co-conservation of particular cysteine residues suggests that these cysteine residues participate in a disulfide bond either within the same molecule or across different molecules (“1–1” and “2–2”). Six cysteine residues (c1 through c6) are invariant; these are also highly conserved across all of the homologous retroviral *env* genes (Figure 3A). In general, the C-terminal domain of Iris is more conserved than the N-terminus among orthologs, and between Iris and retroviral envelope proteins. Some residues at the C-terminus, after the predicted membrane-spanning peptide, are also highly conserved (PLLEK amino acid residues). Based on bioinformatic predictions and by analogy to retroviral envelope proteins, this represents the cytoplasmic tail of Iris, and suggests that this may participate in either the physical anchoring of Iris at the cell membrane, or some downstream signal transduction.

**Figure 6:**
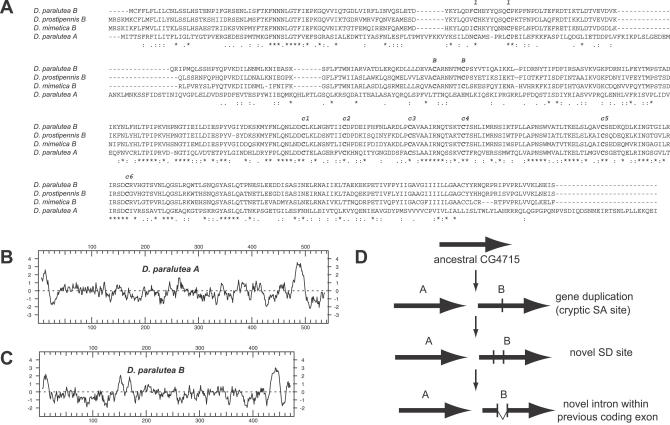
*Iris* Paralogs in the takashii/suzukii Species Groups (A) An alignment of representative Iris-A and Iris-B proteins from the takahashii/suzukii species groups is shown. Iris-A and Iris-B proteins are highly similar to each other. Notable differences include pairs of cysteine residues that are conserved in the B lineage (indicated with “B”), but not in A. The B lineage also has a shorter cytoplasmic tail and is missing several residues (PLLEK amino acid residues) that are invariant in the A lineage. In addition, an internal segment of the Iris-A protein is lost from the Iris-B protein, by virtue of this genomic sequence becoming an intron (confirmed by RT-PCR). (B and C) Hydropathy plots of representative Iris-A and Iris-B proteins show that the overall architecture of the two proteins is largely unaffected by the differences between the two lineages. (D) A hypothetical model for the origin of the divergent *Iris-B* gene starts with the tandem gene duplication. A cryptic SA site is encountered by mutation, but this can be neutrally maintained. However, the simultaneous occurrence of an SD site activates the SA site and leads to a portion of the coding exon being spliced out from the mature message. If this is deleterious, the SD-SA combination is culled out by selection. However, in rare cases, like the *Iris-B* gene, this could lead to a novel functional gene that is favored by selection. Subsequently, the SD and SA sites are maintained by purifying selection.

One feature that is almost universally conserved among retroviral envelope proteins is a furin cleavage site followed by a hydrophobic peptide that represents the fusion peptide. Surprisingly, we found that the Iris protein in *D. melanogaster* lacks the central furin cleavage site and fusion peptide found in all *env* genes capable of mediating infection. We investigated when this cleavage site degenerated on the *Iris-env* phylogeny (Figure 3A). We employed a MAST search [38] using a position-specific scoring matrix constructed from a subset of retroviral homologs as previously described [20]. As a positive control, we used retroviral and baculoviral *env* genes where we knew that the furin cleavage site was conserved. For a negative control, we used homologous baculoviral genes where the furin cleavage site has been shown to have degenerated [39]. We queried three Iris proteins (from *D. melanogaster, D. ananassae,* and *D. pseudoobscura*), one domesticated mosquito gene (from *A. gambiae*), and the *env* gene from the *Kanga* retroviruses using this consensus. Using this strategy, we find that both *Kanga* retroviruses and the domesticated envelopes from mosquito genomes have a conserved furin cleavage and fusion peptide (E-value < 0.001), while this site is not conserved in any of the Iris proteins (E-value > 10). This suggests that the fruit fly and mosquito domestication events differ both chronologically and qualitatively, and that this cleavage site has been lost in the Iris lineage. This loss of cleavage is especially noteworthy since other architectural features, including several conserved pairs of cysteine residues (c1 through c6) presumed to be necessary for membrane fusion ability and post-cleavage interactions between the SU and TM domains, are still conserved [22] (Figure 5). This suggests that while these features are essential for membrane fusion, they may also serve another function.

### A Second *Iris* Gene in the *takahashii/suzukii* Species Groups: A New Mode of Neofunctionalization?

All *Drosophila* species that we have investigated in the *Sophophora* subgenus (Figure 2B) possess an *Iris* ortholog in a syntenic location. Surprisingly, the *takahashii/suzukii* species groups have two genes that are found in tandem orientation (Figure 2A). We have shown that the first of these *(Iris-A)* represents the true ortholog while the second *(Iris-B)* is paralogous (Figure 2C). At first glance, the second gene *(Iris-B)* appears to be a pseudogene. All other *Iris* orthologs are intron-less genes. Based on this expectation, *Iris-B* (which is the same length as *Iris-A*) has frameshifts and nonsense codons. However, when we did RT-PCR on this gene in *D. lutescens* and *D. prostipennis,* we found that these genes had spliced out an intron of ~70 nucleotides. Removing this intron now recapitulates an open reading frame that is highly homologous to that of *Iris-A.* We found that the splice acceptor (SA) and splice donor (SD) sequences are invariant, and we conclude that all *Iris-B* genes possess a single intron. An amino acid sequence comparison of representative *Iris-A* and *Iris-B* genes is presented in Figure 6A. Once again, the C-terminal half of the gene is well conserved (including c1 through c6, highlighted in Figure 5), with more variation in the N-terminus. Hydropathy plots (Figure 6B and 6C) illustrate that despite the loss of exonic sequence, the overall architecture of the *Iris-B* proteins is largely unaltered. Some differences are apparent, however. For instance, *Iris-B* lacks the conserved residues at the C-terminus of *Iris-A* (PLLEK amino acid residues). Additionally, *Iris-B* has some conserved pairs of cysteine residues that are not found in *Iris-A,* suggesting that *Iris-B* now operates under altered selective constraints. This may be partly responsible for the “early branching” of the B lineage in the *Iris* phylogeny (Figure 2C).

Their maintenance since the evolutionary origin of the *takahashii/suzukii* groups suggests that the *Iris-B* genes are evolving under selective constraints. Following gene duplication, a duplicate gene can suffer three fates: non-functionalization (degeneration of function), neofunctionalization (new function), or subfunctionalization (assortment of ancestral functions). We cannot distinguish between the latter two possibilities. Nonetheless, the striking differences in *Iris-B* compel a hypothetical parsimonious model (Figure 6D) as to how the differences arose. Cryptic SA sites likely occur and are lost neutrally, but the simultaneous gain of an SD site leads to a selective decision. If the spliced product is non-functional, the SD-SA combination is lost. However, if there is a sufficient selective advantage for the truncated protein, then the SD-SA combination will sweep through the population and be maintained by purifying selection. Previously, there has been at least one well-documented instance of a previously intronic or intergenic sequence becoming exonic and a previously exonic sequence becoming a promoter (the *Sdic* gene in *D. melanogaster* [40]), but the de novo “invention” of an intron in what previously was exclusively exonic sequence appears to be a novel finding. The scenario we have presented in Figure 6D is simple but likely to be quite rare. However, unlike the much more frequent event of intron transposition, it is conceivable that intron invention may have contributed extensively to the gain of new protein functions.

### Molecular Evolution of *Iris* in *Drosophila* Species

Most retroviral insertions into the host genome are either detrimental or selectively neutral. Therefore, upon insertion into host genomes, these proviral genes start decaying due to mutation. However, retroviral genes that are beneficial to the host genome can be domesticated; these genes can evolve either under purifying or positive selection. In the first scenario, the newly domesticated gene now carries out a housekeeping function, and selective pressures cull out deleterious mutations, including the majority of those that change the amino acid sequence. The mammalian domesticated *syncytin* gene falls into this category [12,14,15]. On the other hand, the host could also recruit a retroviral gene to protect itself from future rounds of infections, as murine genomes appear to have done with the domestication of a *gag* gene, *Fv1* [11,41], or an *env* gene, *Fv4* [42]. In either scenario, i.e., housekeeping or defense, the domesticated gene is likely to be well conserved because it confers a selective advantage, but the selective pressures are quite distinct and likely to discriminate among possibilities of function. For instance, in the latter host defense scenario, the newly domesticated gene might evolve rapidly at the amino acid level due to selective pressures to keep pace with rapidly evolving infectious agents, as is the case for *Fv1* [41].

**Figure 7:**
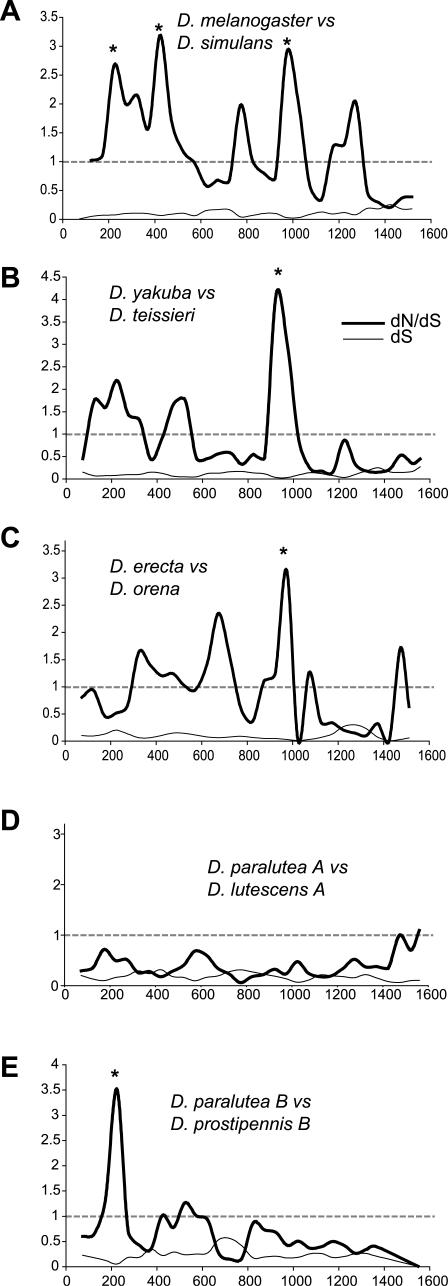
Sliding Window dN/dS Analyses of Different *Drosophila Iris* Genes We have chosen non-overlapping sets of the *Drosophila* species to do a pair-wise analysis of dN compared to dS. We present a sliding window analysis (window size 150 base pairs, slide of 50 base pairs) of dS and the dN/dS ratio (*y*-axis) plotted against nucleotide position (*x-*axis). Under neutrality, a dN/dS ratio of 1 is expected (dashed lines). We present a comparison of (A) *D. melanogaster* versus *D. simulans,* (B) *D. yakuba* versus *D. teissieri,* (C) *D. erecta* versus *D. orena,* (D) *D. paralutea* A versus *D. lutescens* A*,* and (E) *D. paralutea* B versus *D. prostipennis* B. In all these comparisons except (D), at least one window where dN/dS significantly exceeds 1 is seen (indicated by asterisks; significance tested by simulations in the K-estimator program [43]).

What selective constraints have shaped *Iris* evolution? Since *Iris* is a host gene related to retroviral *env* genes, we were interested in investigating the selective pressures under which it has evolved. We compared synonymous (dS) and non-synonymous (dN) nucleotide changes in five, non-overlapping pair-wise comparisons across the *Drosophila* phylogeny [43]. These results are presented in Figure 7 and highlight the variable nature of selective constraints, which have acted on *Iris* in the course of its evolution in *Drosophila* species. We find several windows where dN/dS significantly exceeds 1, but the location of these windows is variable from one pair-wise comparison to the next. In the case of the *Iris* paralogs in the *takahashii* species group, we find no evidence of positive selection in the *Iris-A* comparison (Figure 7D), but a significant window in the N-terminus of *Iris-B* (Figure 7E). This could simply reflect stochastic differences, but it is intriguing that the *Iris-A* comparison is the only one in our set that did not show any windows where dN/dS significantly exceeds 1.

**Figure 8:**
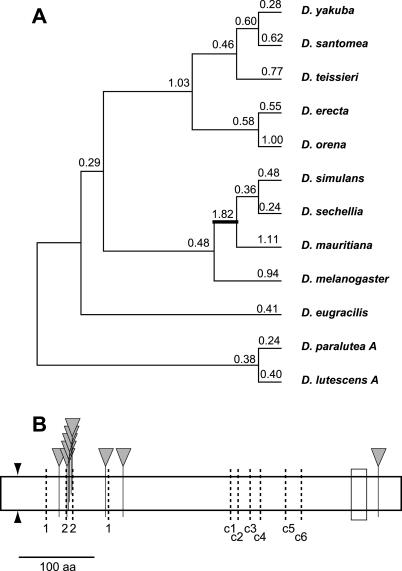
PAML Analyses of *Iris* Evolution (A) A free-ratio model for *Iris* evolution in *Drosophila* is presented with numbers above branches indicating (whole gene) dN/dS ratios estimated for each individual branch. Only the lineage leading to the sibling species *D. mauritiana, D. sechellia,* and *D. simulans* (thick line) has a dN/dS ratio that appears to be greater than 1. When this value of dN/dS = 1.82 was compared against the neutral expectation of 1, the higher value fit the data marginally better (*p* < 0.08). (B) Individual residues highlighted by PAML analyses as having being subject to recurrent positive selection are shown by inverted triangles. Also schematized are the signal peptide cleavage site (arrowheads) and C-terminal hydrophobic peptide (box). Dark, dashed lines indicate the ten cysteine residues (1–1, 2–2, c1 through c6) highlighted in Figure 5. We note that most of the residues identified at high confidence appear to cluster around the 2–2 pair of cysteine residues, suggesting a functional interaction surface here [46].

We also performed a maximum likelihood based analysis of selective pressures acting on *Iris* using the PAML and random effects likelihood (REL) and fixed effects likelihood (FEL) programs [44,45]. We chose only a closely related set of full-length *Iris* orthologs (12 total up to *D. eugracilis*) for this purpose, to minimize the number of gapped positions in the alignment. We excluded all positions with gaps to avoid any ambiguities in alignments. Notably, these gapped regions had the maximum variability in sequence. Results from these analyses are shown in Figure 8A and Table 1. A whole-gene assignment of dN/dS ratios to the different branches of the *Iris* phylogeny is shown in Figure 8A. Only three branches were shown to have a dN/dS greater than 1. This is not surprising because domains subject to purifying selection (where dN/dS is less than 1) can mask the signal of windows of positive selection such that the overall dN/dS in the gene does not exceed 1. In spite of this, we found that the lineage leading up to the sibling species *D. mauritiana, D. simulans,* and *D. sechellia* had a dN/dS ratio of 1.82. Using PAML comparisons in which this branch was fixed at dN/dS = 1 versus dN/dS = 1.82, we found weak evidence that positive selection occurred on this branch (highlighted in Figure 8A; 2ΔlnL = 3.1 and 1 degree of freedom, *p* < 0.08) despite the fact that the whole gene was being analyzed.

**Table 1:**
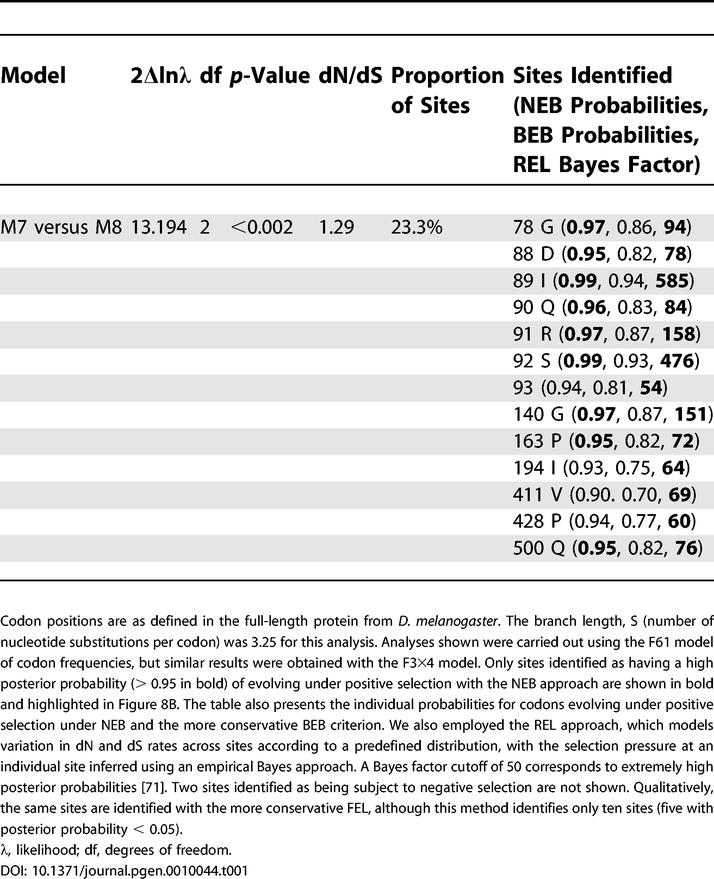
PAML and REL Analyses of *Iris* in *Drosophila* [44]

A whole gene dN/dS ratio comparison can fail to identify specific domains or residues subject to positive selection. We investigated this latter possibility on the multiple alignment of *Iris* from 12 *Drosophila* species using a comparison of NSsites model M7 (a beta distribution with no positive selection) and model M8 (a beta distribution with positive selection permitted). We find that model M8, which allows one class of codons to have allowed under positive selection, fits the data significantly better (Table 1, *p* < 0.002). Thus, we conclude that *Iris* has been subject to positive selection through this period of *Drosophila* evolution. This analysis also highlights a few residues as being repeatedly subjected to positive selection (posterior probability > 0.95 in Table 1). There is remarkable congruence between these results and those obtained from a similar REL analysis and the more conservative FEL analysis (Table 1). Of the nine residues that were identified by the PAML analysis over the entire protein (~ 500 residues compared), six are clustered within 15 amino acids around the 2–2 pair of cysteine residues (Figures 5 and 8B). We have previously tested “patches” of positive selection similarly identified by PAML analyses in the retroviral defense gene *TRIM5α* and have shown that they represent interaction interfaces between host and viral proteins [46]. These analyses suggest that the 2–2 pair of cysteine residues may encode a similar interaction interface.

To investigate the effects of positive selection on standing genetic variation, we sequenced *Iris* from a variety of strains of *D. melanogaster* (14 strains) and *D. simulans* (17 strains) to carry out population genetic analyses. Using the McDonald-Kreitman test, we first compared fixed interspecies differences to intraspecific polymorphisms at replacement and synonymous sites [47]. Fixed Rf:Sf changes between the two species are 77:25, while the polymorphic Rp:Sp ratio is 90:36. These values are not significantly different from each other (*p* ~ 0.5). Polarizing changes to just the *D. melanogaster* lineage (40:17 versus 44:17) or just the *D. simulans* lineage (49:16 versus 46:21) also did not reject the null expectation. One potential source of discordance between the dN/dS and the McDonald-Kreitman test results could be strong selective pressures acting on the intraspecific polymorphisms, compared to interspecific divergence. This could suggest, for instance, that the bulk of the dN/dS signal observed in Figure 7A was in fact due to intraspecific polymorphisms. However, we confirmed that this was not the case by reconstructing the hypothetical ancestor of all *D. melanogaster* and all *D. simulans* strains and performing a pair-wise dN/dS comparison, which is practically identical to Figure 7A (unpublished data).

### *Iris* and the Phylogeny of *D. simulans* Sibling Species

**Figure 9:**
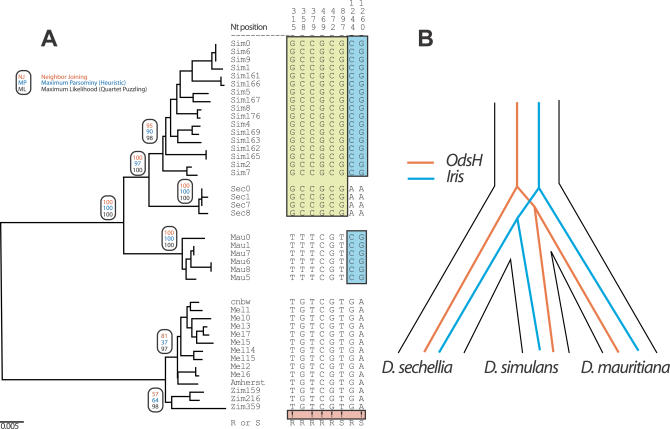
*Iris* Phylogeny in Closely Related Species (A) Phylogenetic analysis of *Iris* coding regions from different strains of *D. melanogaster, D. simulans, D. sechellia,* and *D. mauritiana,* the latter three species believed to have diverged less than half a million years ago [51]*.* Based on distance, parsimony or likelihood methods (bootstrap values indicated in ovals), the phylogeny clearly separates the three species. This is largely due to six sites that are “unambiguous” as far as phylogenetic information is concerned, indicated with “!.” An unambiguous site is defined as one in which the same derived nucleotide is found fixed in two of the three species (e.g., *D. simulans* and *D. sechellia*), whereas the third species (e.g., *D. mauritiana*) is fixed for the ancestral nucleotide, corresponding to the out-group, *D. melanogaster.* (B) *Iris* is only the second known gene to inform about the phylogeny of the three sibling species *D. simulans, D. sechellia,* and *D. mauritiana* with statistical significance*.* In the *Iris* phylogeny, *D. mauritiana* branched earliest while previously, *D. sechellia* was found to branch earliest*.* This suggests that speciation events' chronology among these three species is more complicated than suggested previously [52].

Positive selection may have had a strong impact on *Iris* evolution even in closely related species, due to species-specific infections by mobile elements. Horizontal transfers of DNA-mediated transposons and LTR-retrotransposons [28,48–50] can lead to species-specificity of transposon propagation. These selective pressures could be predicted to lead to the rapid fixation of *Iris* polymorphisms in a species-specific manner, which might subsequently resist introgression of alleles from other species because of constant selective pressures. We tested these possibilities by comparing *Iris* sequences from several strains of *D. simulans, D. mauritiana,* and *D. sechellia* since these species appear to have the most striking signature of positive selection (Figure 8A). These three species are believed to have separated less than 500,000 years ago [51]. In our phylogenetic analysis (Figure 9A), *Iris* sequences from each species form their own exclusive clade to a high degree of statistical support, in large part due to six nucleotide differences that are unambiguously diagnostic for branching order within these three sibling species.

The ability to phylogenetically separate these three species has only been seen previously for the *Odysseus* homeobox *(OdsH)* gene [52] that has been proposed to play a role in hybrid sterility [53]. The difficulty in resolving these relative recent speciation events is likely to result from the persistence and possibly introgression of ancestral alleles following speciation [51,52]. Indeed, since only speciation genes would be able to resist the effects of introgressed alleles from other species, it has been previously suggested that only these would have the required resolution to trace the exact chronology of reproductive isolation among recently diverged species. Based on the *OdsH* gene, the case has been made for allopatric speciation among the sibling species *D. simulans, D. mauritiana,* and *D. sechellia,* with *D. sechellia* branching first [52].

Our results call into question the generality of these previous conclusions. While *Iris* also resolves the phylogeny to almost the same degree of certainty, the chronology of events traced by *Iris* are different from those traced by *OdsH.* Thus, in the case of *OdsH,* six “unambiguous” sites indicated that *D. sechellia* was the out-group, while one site indicated that *D. simulans* was the out-group [52]. In the case of *Iris*, five sites (all in the N-terminus) indicate that *D. mauritiana* was the out-group species while one (the most C-terminal) indicates that *D. sechellia* was the out-group. We suggest that it is likely that all these phylogenetic reconstructions simply reflect the fact that a recent episode of positive selection affected only two out of three species, rather than the true chronology of reproductive isolation. Notably, *OdsH* is under strong positive selection between *D. mauritiana* and *D. simulans,* and its phylogeny groups these two species [52,53]. Similarly, there appears to be clear evidence that *Iris* is significantly diverged because of a species-specific selective pressure.

An important caveat is that both these genes reside on different chromosomes: *OdsH* on the X and *Iris* on 2L. Divergent selective regimes could have led to independent, species-specific chromosomal “speciation” events, although it is difficult to imagine how this could have been achieved in strict allopatry if they occurred simultaneously. Alternatively, the *OdsH* and *Iris* phylogenies could reflect temporal differences, with positive selection acting on *OdsH* at the speciation bottleneck that occurred in allopatry, while a different episode of positive selection acted on *Iris* subsequently. Interestingly, we find that *Iris* also separates the Zimbabwe strains from the cosmopolitan strains of *D. melanogaster* (Figure 9A phylogeny; unpublished data), consistent with known premating isolation between these populations [54].

## Discussion

The evolutionary origin of viruses has long fascinated evolutionary biologists. Are they remnants of an ancestral lifestyle, or more recent escapees from traditional genomes [55]? The *env* genes of retroviruses are an important key to unlocking this conundrum; as first suggested by Howard Temin [56], their acquisition is the single event that allows previously genome-bound retrotransposons to adopt an infectious lifestyle. The genes that confer this ability appear to have been very desirable for eukaryotic genomes. In particular, the *syncytin* genes have been acquired in two mammalian lineages, while *Iris*-like genes have been acquired in two insect lineages. However, there are significant differences between the *syncytin* and *Iris* domestication events. First, the *syncytin* genes show a signature of purifying selection in primates, consistent with their domesticated role in placental function [15]. *Iri*s*,* on the other hand, appears to be an active participant in an ongoing genetic conflict as evidenced by the signature of positive selection. Second, the *syncytin* gene has retained the same architecture of the ancestral retroviral *env* gene including the SU/TM furin cleavage site, since it still carries out the ancestral membrane fusion function [12,14]. *Iris,* on the other hand, has degenerated this cleavage site, suggesting that *Iris's* current function does not require membrane fusion. Third, while *syncytin* clearly derived from an endogenous retrovirus, the donor retroviruses appear to be extinct, especially in the human genome. However, the *Kanga* retroviruses appear to be active, which may greatly aid studies on this interesting domestication of a retroviral *env* in an organism with more facile genetics.

Are the selective pressures on *Iris* unique? We know of two other cases of proviral *env* genes domesticated for host defense: *Fv4* and *Rmcf.* Neither has been investigated for selective constraint. However, in the case of both *Fv4* and *Rmcf* [42,57], the mode of defense is by the domesticated *env* gene blocking the receptor required for retrovirus entry [58,59]. Under this scenario, unless the receptor is subject to positive selection, the domesticated gene does not have a “moving target” and is not expected to be subject to positive selection. Indeed, the defense function of *Fv4* and *Rmcf* may involve the stable co-evolution of the receptor and the domesticated ligand. *Iris,* on the other hand, is subject to positive selection, suggesting that its mode of action is likely to be directly at a protein–protein interaction surface with its antagonist [46]. Thus, we predict that *Iris* action is likely to be distinct from the receptor blockade mechanism.

**Figure 10:**
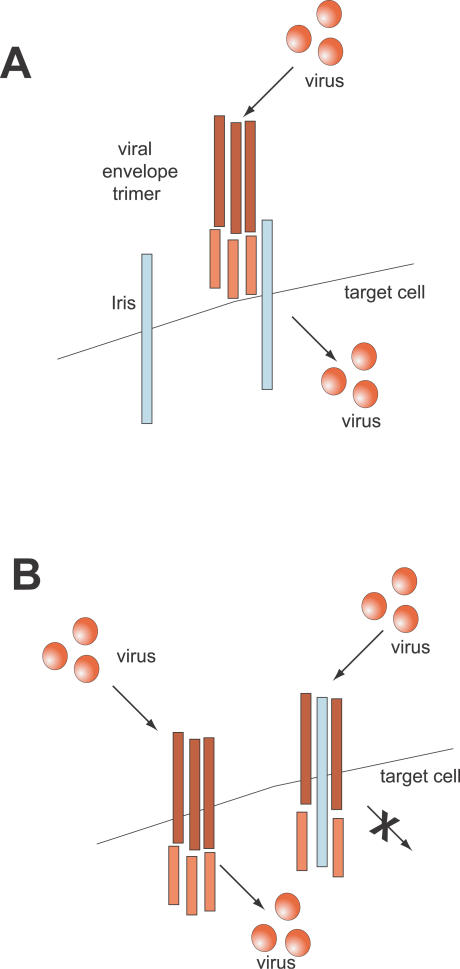
Two Hypothetical Models to Explain Positive Selection of *Iris* (A) Under the first model, *Iris* has been domesticated for a role other than host defense. As part of this housekeeping function, Iris proteins reside on the cell surface, where they can be recognized as receptors by viral envelope proteins. Variants of *Iris* that cannot be recognized by the viral envelopes have a selective advantage. (B) A second model considers the possibility that *Iris* can act as a dominant negative agent that counteracts retroviral envelope trimers (red) from mediating infection. In this scenario, viruses encode for envelope trimers that can be cleaved into the SU ligand interaction and TM membrane fusion domains. In the absence of *Iris,* or if *Iris* lacks the specificity to bind the envelope trimers, the viral envelopes can mediate infection of the target cell. However, if the Iris protein (blue) can bind the viral envelopes and arrest the membrane fusion step, then the host defends against the viral infection. In this scenario, *Iris* directly acts as a host defense protein. Note that in both scenarios, *Iris* is predicted to be subject to positive selection (to decrease virus binding in the first model, and to increase virus binding in the second).

What genetic conflict could *Iris* be subject to? Previously, there has been one case of positive selection of a viral gene that was recruited as an inhibitor of subsequent infections. The *Fv1* restriction factor that guards against murine retroviral infections is a “domesticated” *gag* gene from a lineage of retroviruses [11] that has been proposed to be subject to positive selection in murine genomes [41]. Based on our finding of positive selection, and the precedent of the *Fv1* gene, we propose that *Iris* has been recruited as a host gene specifically to defend adults against recurrent invasions by retroviruses and baculoviruses, which share a homologous *env*. Two hypothetical scenarios by which this defense could be achieved are schematized in Figure 10.

In the first model (Figure 10A), *Iris* is present on cell surfaces as part of a housekeeping function, as is the case for *syncytin.* But by virtue of its homology, it continues to act as a receptor for retroviruses. Under this scenario, the positive selection on *Iris* would cause it to avoid interacting with retroviral envelopes. Whether *Iris* has a housekeeping function can be directly tested with flies carrying mutations in the *Iris* gene. Under the second model (Figure 10B) *Iris* serves as a dominant negative inhibitor of retroviral trafficking. Since the *Iris*-encoded protein is expected to largely share the same architecture as the retroviral envelope proteins, it is expected to form multimers with the retroviral encoded envelope proteins. However, if the protein encoded by *Iris* is not cleaved, these may form multimers with retroviral envelopes that are incapable of mediating infection. In this scenario, the positive selection of *Iris* would act to improve recognition of retroviral envelope proteins to trap them in defective multimers (Figure 10B), while the latter would evolve away from this inhibitory interaction. We favor this second model because it provides a rational hypothesis for why the furin cleavage site has not been conserved. Under this model, we expect that *Iris* could defend against either horizontal transfers or germline transposition events. Germline tissues (ovaries and testes) are primarily where genome-bound retroelements need to transpose in order to increase their copy number within the genome. Gypsy-like retroviruses appear to infect the female oocyte [60], and recent studies indicate that this infection does not require the retroviral *env* genes [61–63]. However, these retroviruses have also been shown to be able to horizontally transfer to new hosts within the same species [64] and possibly to new species [65], and this activity depends on retroviral *env* activity.

Both models presented in Figure 10 are predicted to result in positive selection on *Iris;* genes subject to constantly antagonistic interactions (the “Red Queen” hypothesis [66]) are frequently subject to positive selection affecting the protein–protein interaction interface [46]. Our results raise the possibility that a number of retrovirus-derived “fossils” that can be found in many genomes, including our own [9], may represent new and old recruits in an ongoing battle for evolutionary supremacy. Such recruitments are easier to identify in genomes like *Drosophila,* where genes that are not under selection are quickly abraded [29], rather than in mammalian genomes, where pseudogenes can survive for tens of millions of years. In both cases, only detailed investigations of function or selective constraint can ascertain whether a retroviral remnant has been functionally retained, or is simply a paleontological relic of a past infection.

## Materials and Methods

### *Drosophila* strains.

*Drosophila* strains used in this study were obtained from the *Drosophila* Species Stock Center (Tucson, Arizona, United States), except for the Zimbabwe strains of *D. melanogaster* that were a gift from Y. Chen and W. Stephan.

### PCR.

PCR was used to amplify the *Iris* coding region from *Drosophila* species using degenerate primers designed to *Iris, CG4715degF*: 5′- CTGGTGGACACCGAAACACCNTACYTNGG-3′, and to a conserved gene found downstream and in opposite orientation to *Iris (CG455*2*)*-primer *CG4552degF*: 5′- GCGACCTCATCACGTTYAARTAYGG-3′ (Figure 1A). This pair of primers enabled the amplification of the 3′ end of the *Iris* coding region and the design of species-specific primers. For all strains from *D. melanogaster,* and sibling species *D. simulans, D. mauritiana,* and *D. sechellia,* we employed specific primers *CG4715eATG*: 5′- AACGATCACCTCTACAAGCGAAAGATG-3′, and *CG4715R2*: 5′- GAAGACTGGTTCCGTATGGCCGC-3′ in forward and reverse orientations, respectively, to get the complete coding *Iris* sequence. In the case of the other *Drosophila* species, we employed a forward primer 500 base pairs upstream of *CG715*: 5′- CACTTCGACTGTTCTGAATGAACTGACG-3′ to obtain nearly the entire coding region, in conjunction with primers designed specifically to the 3′ end of the particular *Iris* gene from that species. Specific primers to *D. pseudoobscura* and *D. ananassae* were made based on the draft sequences of the genome from the two species. The *A. gambiae* sequence was directly obtained from the *Anopheles* genome sequence, while the *A. aegypti* sequence was reconstructed using synteny to *Anopheles,* from the database of trace sequences. Sequences of the *Kanga* and *roo* retroviruses were obtained from the ongoing genome sequencing efforts in 12 *Drosophila* species. Most products were directly sequenced using ABI Big-Dye sequencing. In cases where PCR products were too weak to be directly sequenced, they were cloned using TopoTA cloning kits (Invitrogen, Carlsbad, California, United States) and then sequenced using vector-specific primers: at least four separate clones were sequenced for each PCR product. All sequences obtained or annotated in this study have been deposited in GenBank (http://www.ncbi.nlm.nih.gov/Genbank).

### RT-PCR.

RT-PCR analysis was carried out on pools of polyA *D. melanogaster* RNA that were a gift from S. Parkhurst (Fred Hutchinson Cancer Research Center) using primers *CG4715eATG* and *CG4715R2* (described above) using the SuperScript One Step RT-PCR System from Invitrogen.

### Northern analysis.

Northern analysis was also carried out using a blot containing the same pools of polyA RNA, using *D. melanogaster Iris* gene PCR fragment (*CG4715eATG* and *CG4715R2* primers) as probe. For the tissue-specific RT-PCR analysis, individual flies were dissected for ovaries, testes, carcasses, and heads. RNA was isolated using the Qiagen RNeasy Kit (Valencia, California, United States) and treated with the DNase-Free kit from Ambion (Austin, Texas, United States) to remove trace amounts of DNA. RNA amounts were measured using a spectrophotometer. Roughly equal amounts of RNA were used as template in the individual RT-PCR reactions. As a loading control, and to rule out genomic DNA contamination, a separate RT-PCR was carried out to the *Karyopherin α3* gene using primers 5′- CGTTGAGCTGAGGAAGAACAAGCG-3′ and 5′-GTGGCTGCACGACTCCGTGC-3′, which span an intron, allowing cDNA to be distinguished from genomic DNA. For the *Iris-B* genes from *D. prostipennis* and *D. lutescens*, RNA was isolated from pooled adult male and female flies. RT-PCR was used to validate the intron positions.

### Bioinformatic analyses.

We used PSI-BLAST analyses to obtain all homologous sequences to *Iris (CG4715)* using *Iris, gypsy env,* and *Autographa californica nucleopolyhedrovirus orf23* genes as search seeds, allowing the search up to three iterations. The various homologous sequences obtained by PSI-BLAST and our PCR results were aligned using CLUSTALX [67], eliminating all domains that were not unambiguously aligned in order to get a conservative alignment. Alignments were presented using the MacBoxshade program (written by M. Baron). We then used this alignment to obtain phylogenetic trees using the PAUP* suite of programs [68], employing both neighbor-joining, maximum likelihood, and maximum parsimony (heuristic) searches, followed by bootstrap analyses. The *Kanga* retroviral sequences used in the analysis presented in Figure 3 represent best match hits (using *Iris* as a query) in the individual genomes. Each hit to a retroviral *env* gene was used to analyze the genomic region containing the retrovirus for additional open reading frames, including the *gag*- and *pol*-like genes (used in Figure 3B). We used the SignalP program (version 3.0) to identify putative signal peptide cleavage sites [69].

### Population genetic analyses.

Population genetic analyses were carried out using the DNASP program [70]. We used the program to carry out various tests for positive selection, including the McDonald-Kreitman test [47]. dN/dS ratios were computed in a sliding window using the Kestimator package [43]. Given calculated transition: transversion ratios and G+C content at third positions of codons, 1,000 trials of simulating dN equal to dS were generated. Significant deviations from neutrality (dN/dS ~1) were evaluated by comparing the range of simulated dN values to actual dN [43].

### Maximum likelihood analyses.

Maximum likelihood analyses were performed with Codeml in the PAML software package [44]. Global ω ratios for the tree (Figure 8A) were calculated by a free-ratio model, which allows ω to vary along different branches. To detect selection, multiple alignments were fitted to either the F3 ×4 or F61 models of codon frequencies. Log-likelihood ratios of the data were compared using different site-specific (NSsites) models: M7 (fit to a beta distribution, ω > 1 disallowed) to M8 (similar to model 7 but ω > 1 allowed). The likelihood ratio test is performed by taking the negative of twice the log-likelihood difference between the two models and comparing this to the χ^2^ distribution with degrees of freedom equal to the difference in the number of parameters between the models. In all cases, permitting sites to evolve under positive selection gave a much better fit to the data (Table 1). These analyses also identified certain amino acid residues with high posterior probabilities (greater than 0.95) of having evolved under positive selection under the naïve empirical Bayes (NEB) model (Table 1 and Figure 8B). A more conservative Bayes empirical Bayes (BEB) evaluation of whether codons had evolved under positive selection was also carried out. REL and FEL analyses were carried out using the online server at http://www.datamonkey.org [45,71].

## Supporting Information

### Accession Numbers

The GenBank (http://www.ncbi.nlm.nih.gov/Genbank) accession numbers in this paper are: *Iris-A* sequences (DQ 177366–DQ177418) and *Iris-B* sequences (DQ 185599–DQ 185602).

The FlyBase (http://flybase.bio.indiana.edu) accession numbers in this paper are: CG4715 orthologs (FBgn0031305) and *Anopheles gambiae* homolog of CG4715 (XP_314732).

## Abbreviations

BEB: Bayes empirical Bayes
dN: number of replacement changes per site
dS: number of synonymous changes per site
*env*: *envelope*
FEL: fixed effects likelihood
NEB: naïve empirical Bayes
REL: random effects likelihood
SA: splice acceptor
SD: splice donor
